# RNA editing of nuclear transcripts in *Arabidopsis thaliana*

**DOI:** 10.1186/1471-2164-11-S4-S12

**Published:** 2010-12-02

**Authors:** Yijun Meng, Dijun Chen, YongFeng Jin, Chuanzao Mao, Ping Wu, Ming Chen

**Affiliations:** 1 Department of Bioinformatics, College of Life Sciences, Zhejiang University, Hangzhou 310058, P. R. China; 2 State Key Laboratory of Plant Physiology and Biochemistry, College of Life Sciences, Zhejiang University, Hangzhou 310058, P. R. China; 3 Institute of Biochemistry, College of Life Sciences, Zhejiang University, Hangzhou 310058, P. R. China

## Abstract

**Background:**

RNA editing is a transcript-based layer of gene regulation. To date, no systemic study on RNA editing of plant nuclear genes has been reported. Here, a transcriptome-wide search for editing sites in nuclear transcripts of Arabidopsis (*Arabidopsis thaliana*) was performed.

**Results:**

MPSS (massively parallel signature sequencing) and PARE (parallel analysis of RNA ends) data retrieved from public databases were utilized, focusing on one-base-conversion editing. Besides cytidine (C)-to-uridine (U) editing in mitochondrial transcripts, many nuclear transcripts were found to be diversely edited. Interestingly, a sizable portion of these nuclear genes are involved in chloroplast- or mitochondrion-related functions, and many editing events are tissue-specific. Some editing sites, such as adenosine (A)-to-U editing loci, were found to be surrounded by peculiar elements. The editing events of some nuclear transcripts are highly enriched surrounding the borders between coding sequences (CDSs) and 3′ untranslated regions (UTRs), suggesting site-specific editing. Furthermore, RNA editing is potentially implicated in new start or stop codon generation, and may affect alternative splicing of certain protein-coding transcripts. RNA editing in the precursor microRNAs (pre-miRNAs) of *ath-miR854* family, resulting in secondary structure transformation, implies its potential role in microRNA (miRNA) maturation.

**Conclusions:**

To our knowledge, the results provide the first global view of RNA editing in plant nuclear transcripts.

## Background

RNA editing, defined as any site-specific alteration in RNA sequences including insertion or deletion of nucleotides and base conversion, is an effective way of post-transcriptional gene regulation, and has been widely investigated in animals and plants [1-3]. Different from A-to-inosine (I) editing mediated by adenosine deaminase acting on RNA (ADAR) in mammals [4], C-to-U editing in plants is carried out by pentatricopeptide repeat (PPR) family proteins [3,5]. Previous studies have unraveled the abundance of A-to-I editing in mammalian transcriptomes, and many editing events were demonstrated to be involved in essential biological processes, such as nervous system development [6,7]. However in plants, reports on C-to-U, and less frequently, U-to-C editing, are restricted to mitochondrial or plastid transcripts [3,8,9]. Recently, a large-scale analysis was performed in Arabidopsis and rice to search for candidate editing sites in transfer RNAs (tRNAs) and miRNAs by using small RNA (sRNA) high-throughput sequencing data [10]. However, a global vision of RNA editing in plant nuclear protein-coding transcripts has not been realized.

Here, we carried out an extensive search for potential editing sites in nuclear transcripts utilizing mRNA MPSS and PARE data. The results indicate that RNA editing is an essential RNA-based regulatory layer not only for mitochondrial and chloroplast genes but also for nuclear genes. The data presented could serve as a repository for further analyses, and it will lead to a shift of RNA editing research from well-characterized mitochondrial and plastid transcripts to nuclear transcripts in plants.

## Methods

### Data resources

The Arabidopsis genome information and the GO annotations were retrieved from TAIR (The Arabidopsis Information Resource; release 9, ftp://ftp.arabidopsis.org/home/tair/) [11]. The miRNA information was retrieved from miRBase (release 14, http://www.mirbase.org/cgi-bin/mirna_summary.pl?org=ath) [12]. The MPSS and PARE data were retrieved from the MPSS plus database (http://mpss.udel.edu/at/) and the PARE database (http://mpss.udel.edu/at_pare/), respectively [13,14].

### Clustering analysis

We retrieved MPSS sequences from 17 different libraries with normalized expression data (TPM, transcripts per million). The editing ratio for each editing site was defined as the expression value of all edited reads divided by that of the total reads surrounding the editing site. The single-base sequencing error rate of MPSS was estimated to be ~5.00% [15,16]. Thus, the average single-base sequencing error rate for each error pattern (12 patterns in all) is ~0.42%. To reduce the interference by sequencing errors, only the sites with editing ratios more than 2% in either library were clustered by using Cluster 3.0 [17]. Although the cutoff is arbitrary, the higher percentage surely reflects the higher editing efficiency *in planta*, and the possibility that the editing site may be a feint one generated by sequencing errors can be greatly reduced. The clustering results were visualized by using Treeview [18].

### Other software for data analysis

WebLogo [19] (http://weblogo.berkeley.edu/logo.cgi) was used for sequence conservation analysis. GO::TermFinder [20] was used for GO term enrichment analysis. RNAfold [21] (http://rna.tbi.univie.ac.at/cgi-bin/RNAfold.cgi) was used for pre-miRNA secondary structure prediction. miRU [22] (http://bioinfo3.noble.org/miRNA/miRU.htm) was used for miRNA target prediction.

## Results and discussion

### Editing sites in nuclear transcripts

Fahlman and colleagues revealed ubiquitous RNA modifications in plant tRNAs and miRNAs [10]. However, no research has been carried out to elucidate if RNA editing occurs in nuclear protein-coding transcripts in plants. Here, we focus on one-base conversion in nuclear protein-coding transcripts and pre-miRNAs in Arabidopsis. The MPSS sequences derived from polyadenylation (poly(A))-tailed transcripts were retrieved from the MPSS plus database [13], and the PARE sequences from the 5' ends of miRNA-mediated poly(A)-tailed mRNA decays were retrieved from the PARE database [14]. As nearly all the protein-coding and miRNA genes are transcribed by RNA polymerase II, resulting in poly(A)-tailed transcripts [23,24], the MPSS and PARE data are applicable for this study.

All the short reads were mapped to the pre-miRNAs and the mRNAs of all the protein-coding genes including mitochondrial and chloroplast genes in Arabidopsis. The perfectly matched sequences were removed and the remaining reads were utilized to search for one-base-conversion editing sites. In light of the technological sequencing errors of MPSS and PARE, the protein-coding transcripts and the pre-miRNAs were considered to be edited based on the following criteria as a measure of caution: For each protein-coding transcript, more than two candidate editing sites should be detected and each editing site must be supported by more than five distinct short reads. For each pre-miRNA, the editing site should be supported by more than two distinct short reads. It was estimated that the single-base sequencing error rates were ~5.00% (20-nucleotide (nt) signatures) or ~4.25% (17-nt ones) for MPSS sequencing platform [15,16], and 1.30 ± 0.90% for PARE sequencing [25]. That is, the average sequencing error rates of each error pattern (12 in all) are ~0.42% (20 nt) or ~0.35% (17 nt) for MPSS, and ~0.03—0.18% for PARE. To further assess the reliability of our prediction criteria, the ratio of edited signatures to total signatures including non-edited ones surrounding each editing site was calculated. The ratios range from 12.50% to 100%, and the average ratios are 21.75% for the protein-coding transcripts and 42.05% for the pre-miRNAs (Additional Files 1 and 2). It indicates that a sizable portion of the predicted editing sites are not feint ones generated by sequencing errors.

**Figure 1:**
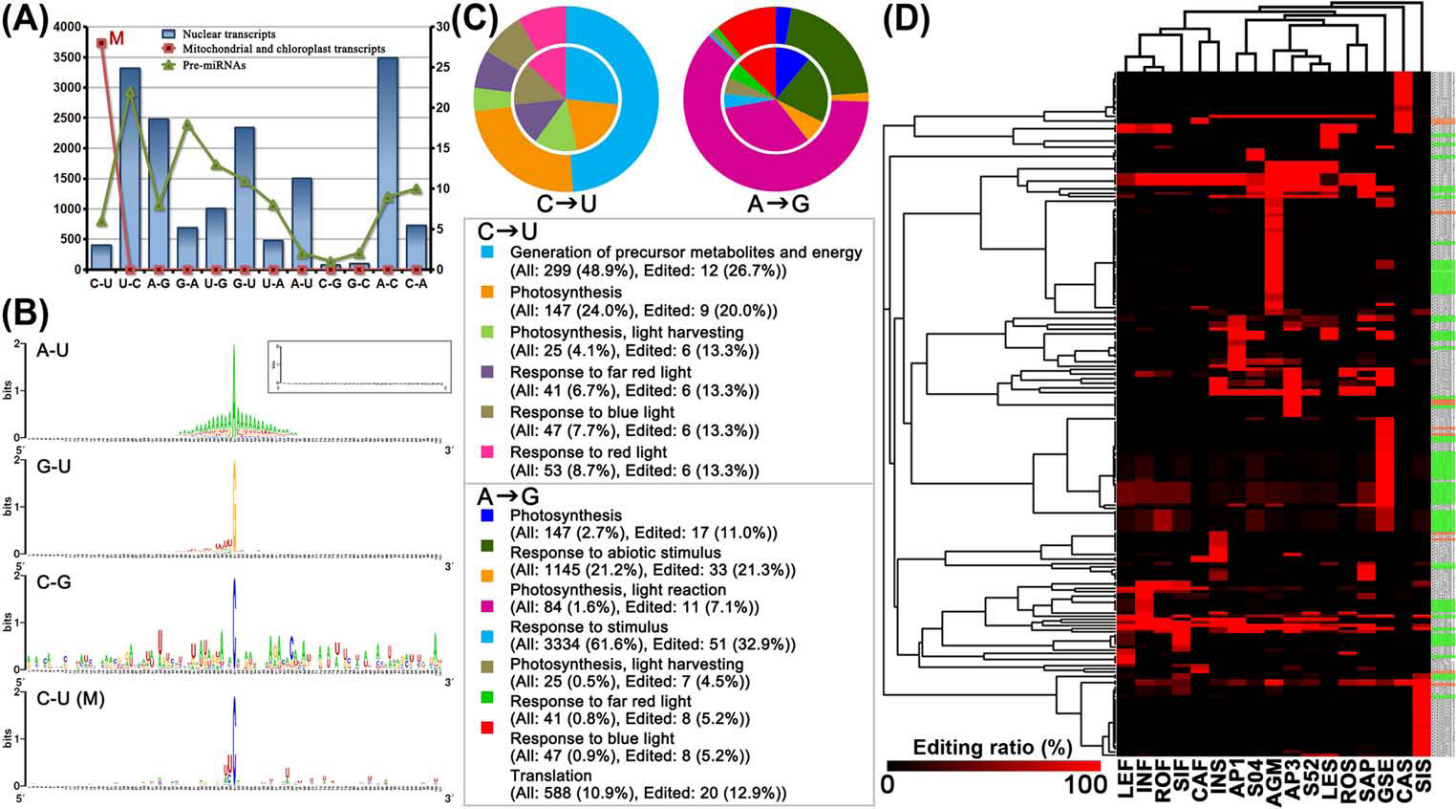
**Overview of RNA editing in plant nuclear transcripts.** (A) Statistics of RNA editing sites in nuclear protein-coding transcripts, pre-miRNAs, and mitochondrial and chloroplast transcripts. The number of editing sites in the nuclear protein-coding transcripts (blue histogram) is measured by left *y* axis and that of the pre-miRNAs (green curve) or the mitochondrial and chloroplast transcripts (red curve) by right *y* axis. The 12 editing patterns are shown on the *x* axis. “M” represents the editing sites in the mitochondrial transcripts and none has been detected in the chloroplast transcripts. (B) Novel elements surrounding the editing sites. The 100-nt sequences (*x* axis) surrounding the editing sites of nuclear protein-coding transcripts were analyzed by using WebLogo. Results of three different editing patterns (A-to-U, G-to-U, and C-to-G) in the nuclear transcripts and C-to-U editing in the mitochondrial transcripts (“M”) are shown. The inset in the upper right corner shows the result for random sequences. (C) GO term enrichment analysis of edited nuclear protein-coding genes. Results of C-to-U and A-to-G edited genes produced by GO::TermFinder are shown. The GO terms, significantly enriched in edited genes (corrected *P*-value < 1.00E-07), are listed at the bottom. “All” represents all the protein-coding genes (the circle outside the pie chart). “Edited” represents the edited protein-coding genes (the inner pie chart). The percentage was calculated by dividing the number of the “All” (or the “Edited”) genes with the certain GO term by the number of all the listed “All” (or “Edited”) genes. (D) Clustering analysis of RNA editing sites. MPSS data from 17 libraries were analyzed. The ratio of the expression value of all the edited reads to that of the total reads surrounding the editing site was calculated. Only the sites with ratios more than 2% were clustered. The ratio values were represented by the color intensity shown at the bottom. On the right, the transcripts with mitochondrion- or chloroplast-related functions are in orange or green shadows respectively. See details of the 17 libraries in Additional File 9: Data S7 or the MPSS plus database (http://mpss.udel.edu/at/).

The result indicates that all 12 RNA editing patterns may exist in the nuclear transcripts, although the number of editing sites in a specific pattern varies widely (Fig. 1A). Previous reports demonstrated that C-to-U conversion was the dominant editing pattern of mitochondrial and plastid transcripts [2,3]. Consistently, our study shows that C-to-U conversion is the exclusive editing pattern in mitochondrial transcripts (Fig. 1A). From another perspective, it reflects that our search criteria are quite reliable, especially in excluding false positive. However, C-to-U editing is not the dominant pattern in the nuclear transcripts analyzed. Instead, U-to-C, A-to-G, G-to-U, and A-to-C are the dominant ones in the nuclear protein-coding transcripts, and U-to-C and G-to-A in the pre-miRNAs (Fig. 1A). A-to-I editing (A-to-G, recognized by sequencing) mediated by ADAR has been extensively characterized in mammals (see reviews in [6,26]), whereas no such editing has been recognized in plants. Our results show that A-to-I editing is likely to be existed in plant nuclear transcripts. However, the ADAR homolog has not been identified in plants yet. Hence, this study will inspire further research to understand the intriguing mechanisms of this peculiar RNA editing pattern in plant nuclear transcripts. Taken together, our preliminary observation (Data S1 and S2) is a valuable repository for further studies on RNA editing in plant nuclear transcripts.

### *Cis*-elements surrounding the editing sites

The 100-nt sequences (Additional File 3: Data S3) surrounding the editing sites (50-nt sequences both upstream and downstream) with specific patterns of nuclear protein-coding genes were submitted to WebLogo [19] for sequence conservation analysis. Conserved elements were detected surrounding the editing sites with certain editing patterns, such as G-to-U and C-to-G. The conserved elements surrounding A-to-U editing sites are quite interesting that the nearer positions, relative to the editing sites, show higher occurring frequency of A (Fig. 1B). However, no obvious sequence conservation was observed surrounding the C-to-U editing sites in the nuclear transcripts, although short conserved elements were present in the mitochondrial transcripts (Fig. 1B and Additional File 4: Data S4). Previous research suggested that a particular *cis*-element surrounding the editing site was required for the recognition by PPR-associated editing enzyme in plants [3]. Our result shows that besides C-to-U editing in mitochondrial and plastid transcripts, other editing with potential conserved *cis*-elements surrounding the editing sites may exist in nuclear transcripts. To better understand the mechanisms implicated in various RNA editing processes, delicate experiments are needed for *cis*-element identification, editing enzyme isolation, and editing site validation.

### Chloroplast- or mitochondrion-related function enrichment of edited nuclear transcripts

For each editing pattern, all the edited protein-coding transcripts compared with whole-genome protein-coding ones were subjected to GO term enrichment analysis. Interestingly, for nearly all the editing patterns, the functionalities of the edited genes are highly enriched in photosynthesis, light response, or energy metabolism (Fig. 1C and Additional File 5: Data S5). Although a number of mitochondrial and chloroplast transcripts have been reported to be edited in plants [2,3], it is surprising that the nuclear transcripts, encoding proteins involved in chloroplast- or mitochondrion-related functions, are more susceptible to RNA editing.

### Tissue-specific editing

We utilized MPSS data from 17 different libraries to investigate the tissue-specific editing patterns. The expression data of each library was normalized to enable cross-library comparison. For each editing site, the ratio of the expression value of all the edited reads to that of the total reads was calculated which represents the editing efficiency. To reduce the interference by sequencing errors, only the editing sites with editing ratios more than 2% in each library were clustered. The clustering result shows that tissue-specific RNA editing, such as in agamous inflorescence, callus, and silique, has been observed in a portion of transcripts (Fig. 1D). The MPSS sequences are composed of 17-nt and 20-nt ones, so we analyzed the two portions separately and tissue-specific editing was still observed in both cases (Additional File 6: Fig. S1).

### Site-specific editing

**Figure 2:**
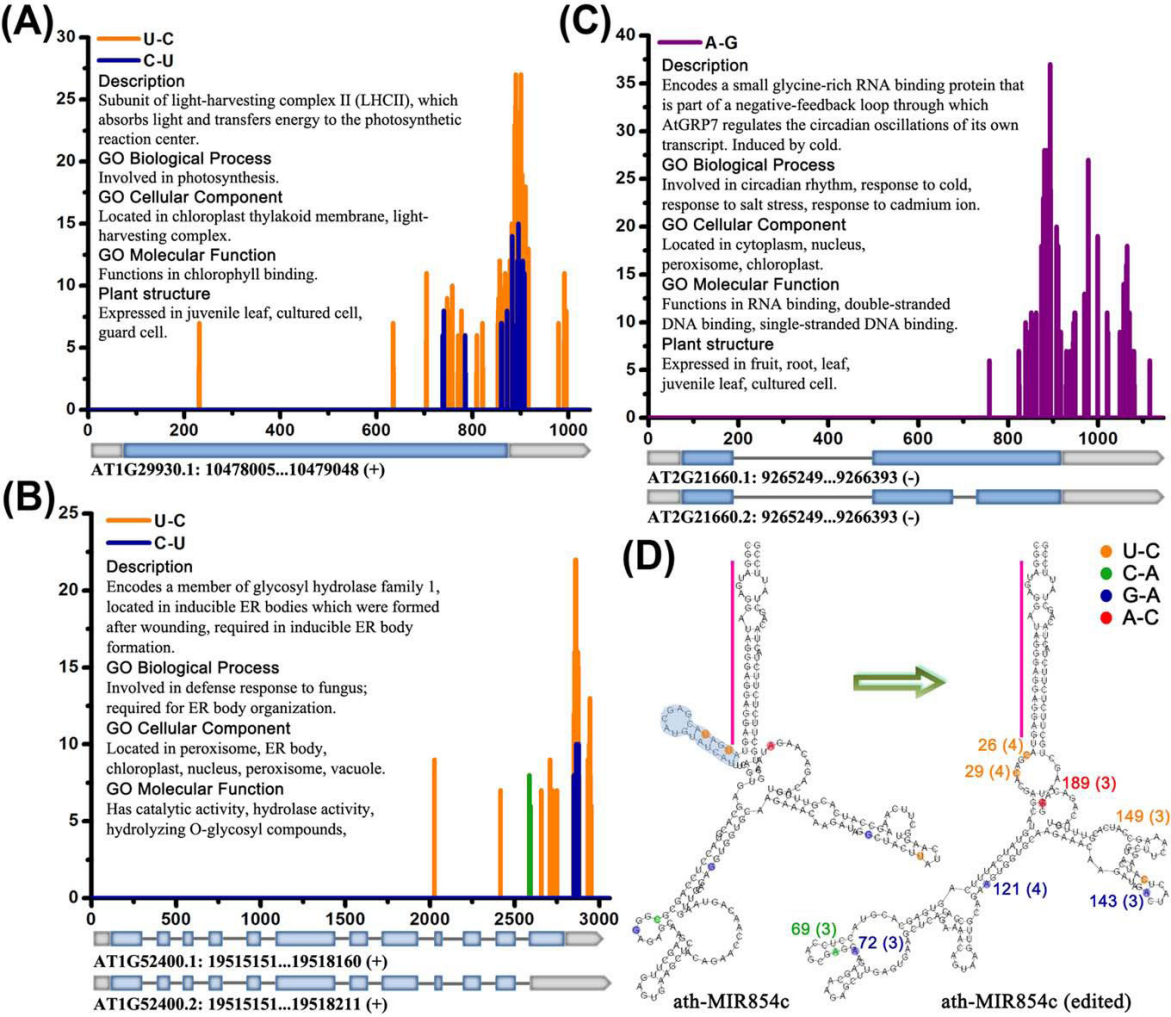
**Specific cases of RNA editing in nuclear transcripts.** (A) U-to-C (orange) and C-to-U (blue) editing in the mRNA of *AT1G29930*. (B) U-to-C (orange) and C-to-U (blue) editing in the mRNAs of *AT1G52400*. U-to-C editing sites that reside only in the mRNA of *AT1G52400.1* are in green. (C) A-to-G (purple) editing in the mRNAs of *AT2G21660*. For (A), (B), and (C), the gene model IDs and the gene annotations are shown. The exons are represented by light blue boxes, the UTR regions by gray boxes, and the introns by lines. The transcript length is measured by *x* axis; *y* axis indicates the number of distinct short-read sequences supporting a specific editing site. (D) Secondary structure transformation of edited *ath-miR854c*. The secondary structure was predicted by RNAfold. Different editing patterns are indicated by different colors; the editing site position and the number of distinct short reads (in the parentheses) supporting this editing site are also shown. The mini stem-loop structure near the main stem region of *ath-miR854c* disappeared after editing is in light blue shadow. Mature miRNA is indicated by a pink bar.

A number of transcripts were subjected to site-specific editing. For *AT1G29930.1* and *AT1G52400.1*, both the C-to-U and the U-to-C editing are highly enriched surrounding the boundaries between the CDSs and the 3’ UTRs, which are also known as translation borders (Fig. 2A and B). Moreover, the C-to-U and the U-to-C editing sites come together, indicating that an amino-group, dissociated from C which further converts to U, could be integrated with the neighboring U that subsequently converts to C. For *AT2G21660.1* and *AT2G21660.2*, A-to-G editing sites are also highly enriched surrounding the translation boundaries (Fig. 2C). The biological means of these site-specific editing events should be further investigated.

### RNA editing involved in new start or stop codon generation and alternative splicing

RNA editing resulted in generation of new start or stop codons has been reported in both humans and plants [27,28] (also see reviews in [6,29]). In this study, a systemic search was performed to identify novel start or stop codons generated by RNA editing in nuclear CDSs. In summary, new start codons are generated predominantly by C-to-U and G-to-U editing, and novel stop codons by G-to-U, A-to-U, and C-to-A editing (Table 1 and Additional File 7: Data S6). These types of editing may produce premature proteins or even new functional ones.

**Table 1:** Start or stop codons generated by RNA editing in nuclear transcripts and statistics of edited nuclear transcripts in Arabidopsis

**Codons generated in nuclear protein-coding transcripts**^a^	Editing patterns												Total No.

	C-U	U-C	A-G	G-A	U-G	G-U	U-A	A-U	C-G	G-C	A-C	C-A	
**AUG**	15	0	1	0	1	13	1	1	0	0	0	3	35
**UAA**	1	0	0	1	0	25	1	18	0	0	0	51	97
**UAG**	1	0	4	3	0	40	0	26	0	0	0	0	74
**UGA**	0	0	3	0	1	69	0	0	0	0	0	11	84

	**Editing patterns**												**Total No. of edited transcripts (pre-miRNAs)**^b^

	**C-U**	**U-C**	**A-G**	**G-A**	**U-G**	**G-U**	**U-A**	**A-U**	**C-G**	**G-C**	**A-C**	**C-A**	

**No. of edited nuclear protein-coding transcripts**	37	136	165	64	65	158	30	107	6	11	199	56	355
**No. of edited pre-miRNAs**	4	9	8	11	13	11	8	2	1	1	7	7	36

^a^ Only the newly generated codons residing in CDSs were included.

^b^ Total number of edited genes or pre-miRNAs is less than the sum of 12 editing patterns because a large portion of protein-coding genes or pre-miRNAs share several editing patterns.

It was reported that certain elements within exons and introns of eukaryotic genes were essential for the splicing of their transcripts, and RNA editing has great potential to affect RNA splicing [6,29-31]. Because all the MPSS and PARE reads were derived from poly(A)-tailed mature mRNAs [13,14], we investigated the RNA editing within the 5’ first and the 3’ last three nucleotides of each exon, both of which will potentially affect RNA alternative splicing. Although only a small portion of nuclear transcripts were found to be edited at either ends of their exons (Additional File 1: Data S1), it suggested that alternative splicing converting pre-mRNAs to mRNAs might be influenced by RNA editing in Arabidopsis.

### RNA editing in pre-miRNAs

Previous research showed that various types of RNA editing occurred in plant tRNAs and mature miRNAs [10]. However, the scene of RNA editing in pre-miRNAs, which may result in secondary structure transformation, has never been unveiled. We searched for potential editing sites in pre-miRNAs (Table 1 and Additional File 2: Data S2) and some interesting secondary structure transformations of edited pre-miRNAs were observed. All the pre-miRNAs of *ath-miR854* family were found to be edited in several sites. Taking *ath-MIR854c* for example, the secondary structure has markedly changed after editing. Notably, a mini stem-loop structure near the main stem region, generating the mature miRNA through Dicer-like 1 (DCL1) cleavage, has disappeared after editing (Fig. 2D). The other three members of *ath-miR854* family were also investigated, and the similar results were obtained (Additional File 8: Fig. S2). Thus, we postulate that the edited versions of *ath-miR854* family members may be much more efficient for mature miRNA production, considering more accessible structures near the main stem regions for DCL1. Another intriguing observation is that the only pre-miRNA in clustering analysis, *ath-MIR161* (MI0000193), is subjected to leaf-specific editing (Fig. 1D and Additional File 6: Fig. S1). Moreover, the mature miRNAs *ath-miR161.1* and *ath-miR161.2* target transcripts belonging to PPR family based on our prediction results generating by miRU. On the other hand, C-to-U editing in mitochondrial and plastid transcripts was reported to be mediated by PPR family proteins [2,3]. Since a few reports has pointed to the involvement of RNA editing in the maturation of miRNAs in metazoans [32,33], our preliminary observations deserve experimental exploration in plants.

## List of abbreviations used

MPSS: massively parallel signature sequencing; PARE: parallel analysis of RNA ends; C: cytidine; U: uridine; A: adenosine; CDS: coding sequence; UTR: untranslated region; pre-miRNA: precursor microRNA; miRNA: microRNA; I: inosine; ADAR: adenosine deaminase acting on RNA; PPR: pentatricopeptide repeat; tRNA: transfer RNA; sRNA: small RNA; TAIR: The Arabidopsis Information Resource; TPM: transcripts per million; poly(A): polyadenylation; nt: nucleotide; DCL1: Dicer-like 1.

## Competing interests

The authors declare that they have no competing interests.

## Authors' contributions

YJM collected high-throughput sequencing data, designed major parts of the experiment, and wrote the paper. DJC performed the analytical research. YFJ and CZM gave many useful suggestions for our analyses, and helped to improve the manuscript. PW and MC conceived of the study, and participated in its design and coordination, and helped to draft the manuscript. All the authors have read and approved the final manuscript.

## Supplementary Material

Additional file 1 - Data S1 Edited sites in protein-coding genes in Arabidopsis (only the editing sites resided in mRNAs were considered) Prediction criteria: more than two candidate editing sites should be present in one transcript and each editing site must be supported by more than five distinct short-read sequences. Click here for file

Additional file 2 - Data S2 Editing sites in pre-miRNAs in Arabidopsis Prediction criteria: each editing site in one pre-miRNA should be supported by more than two distinct short-read sequences. Click here for file

Additional file 9 Data S7 Detailed information of the MPSS and PARE data utilized in this study. Click here for file

Additional file 3 Data S3 100-nt sequences surrounding the editing sites in protein-coding genes, which were utilized for conserved element detection. The random sequences for control analysis are included. Click here for file

Additional file 4 Data S4 Results of searching for conserved elements surrounding the editing sites in protein-coding genes. The results of three replicates of control analysis are included. Click here for file

Additional file 5 Data S5 Result of GO term enrichment analysis of edited protein-coding genes in Arabidopsis. Click here for file

Additional file 6 Figure S1 Clustering analysis of RNA editing sites. Click here for file

Additional file 7 Data S6 Codon variation by RNA editing in nuclear protein-coding genes (only the edited codons resided in CDSs were considered). Click here for file

Additional file 8 Figure S2 Secondary structure transformation of edited *ath-MIR854a, ath-MIR854b,* and *ath-MIR854d*. Click here for file
