# Supplementary material for: RNA editing of nuclear transcripts in Arabidopsis thaliana
Source: BMC Genomics. 2010 Dec 2;11(Suppl 4):S12. doi: 10.1186/1471-2164-11-S4-S12 (PMC3005917; doi:10.1186/1471-2164-11-S4-S12)
Supplement: Additional file 9 — Data S7 Detailed information of the MPSS and PARE data utilized in this study. [file 1471-2164-11-S4-S12-S9.zip › 13-additional-file8/Figure S1.pdf]

Figure S1

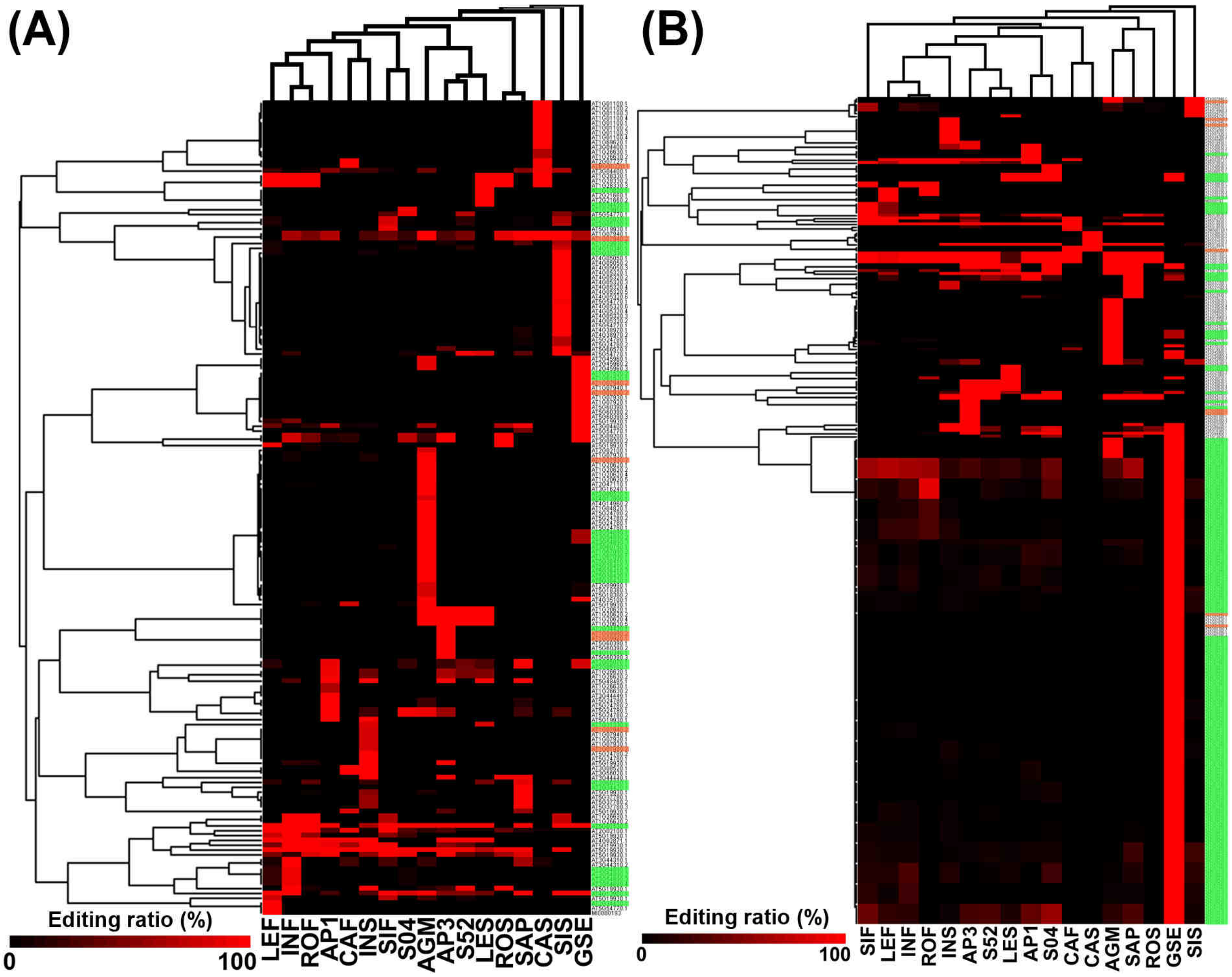

Fig. S1. Clustering analysis of RNA editing sites. The 17-nt (**A**) and 20-nt (**B**) MPSS sequences from 17 different libraries were analyzed separately. For both (**A**) and (**B**), the ratio of the expression value of all the edited reads to that of the total reads surrounding the editing site was calculated. Only the sites with ratios more than 2% were clustered (see details in Materials and Methods in this paper). The ratio values were represented by the color intensity shown at the bottom. On the right, the transcripts with mitochondria- or chloroplast-related functions are in orange or green shadows based on the TAIR annotations (release 9). The 17 libraries are: CAF (callus - actively growing, classic MPSS), CAS (callus - actively growing, signature MPSS), INF (inflorescence - mixed stage, immature buds, classic MPSS), INS (inflorescence - mixed stage, immature buds), AP1 (*ap1-10* inflorescence - mixed stage, immature buds), AP3 (*ap3-6*

inflorescence - mixed stage, immature buds), SAP (*sup*/*ap1* inflorescence - mixed stage, immature buds), AGM (agamous inflorescence - mixed stage, immature buds), SIF (silique - 24 to 48 hr post-fertilization, classic MPSS), SIS (silique - 24 to 48 hr post-fertilization, signature MPSS), LEF (leaf - 21 day, untreated, classic MPSS), LES (leaf - 21 day, untreated), S04 (leaf, 4 hr after salicylic acid treatment), S52 (leaf, 52 hr after salicylic acid treatment), ROF (root - 21 day, untreated, classic MPSS), ROS (root - 21 day, untreated), and GSE (germinating seedlings). Also see details in the *Arabidopsis* MPSS plus database (http://mpss.udel.edu/at/).
