# Supplementary material for: RNA editing of nuclear transcripts in Arabidopsis thaliana
Source: BMC Genomics. 2010 Dec 2;11(Suppl 4):S12. doi: 10.1186/1471-2164-11-S4-S12 (PMC3005917; doi:10.1186/1471-2164-11-S4-S12)
Supplement: Additional file 8 — Figure S2 Secondary structure transformation of edited ath-MIR854a, ath-MIR854b, and ath-MIR854d. [file 1471-2164-11-S4-S12-S8.zip › 13-additional-file9/Figure S2.pdf]

Figure S2

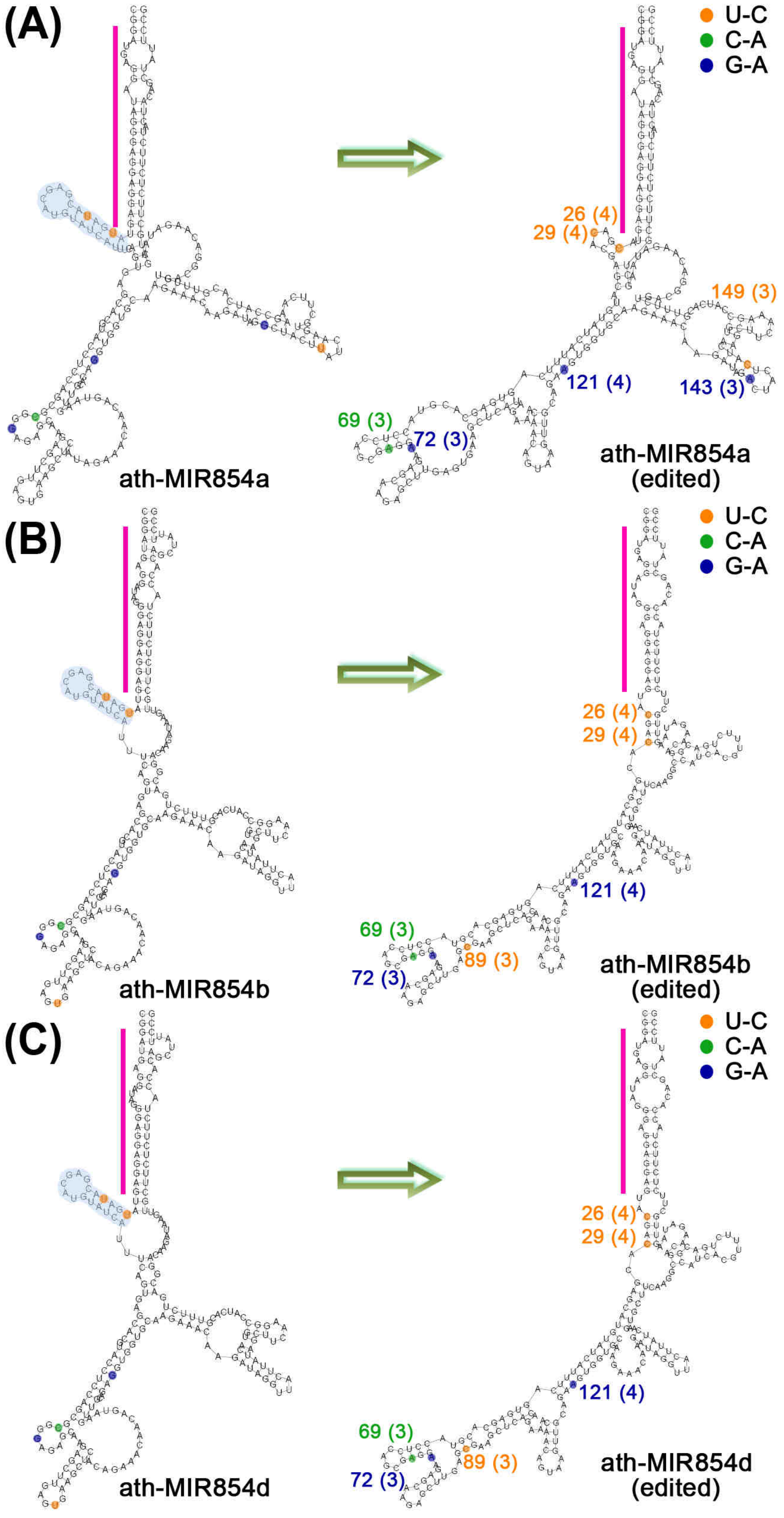
(A)
U-C
C-A
G-A
26 (4)
29 (4)
149 (3)
121 (4)
143 (3)
69 (3)
72 (3)
ath-MIR854a
ath-MIR854a
(edited)
(B)
U-C
C-A
G-A
26 (4)
29 (4)
121 (4)
69 (3)
89 (3)
72 (3)
ath-MIR854b
ath-MIR854b
(edited)
(C)
U-C
C-A
G-A
26 (4)
29 (4)
121 (4)
69 (3)
89 (3)
72 (3)
ath-MIR854d
ath-MIR854d
(edited)

Fig. S2. Secondary structure transformation of edited *ath-MIR854a* (**A**), *ath-MIR854b* (**B**), and *ath-MIR854d* (**C**). For (**A**), (**B**), and (**C**), the secondary structures were predicted by RNAfold (Hofacker, 2003; Gruber *et al.*, 2008). Different editing patterns are indicated by different colors; the editing site position and the number of distinct short reads (in parenthesis) supporting the editing site are also shown. The mini stem-loop structures near the main stem regions of *ath-MIR854a*, *ath-MIR854b*, and *ath-MIR854d* disappeared after editing are in light blue shadows. Mature miRNAs are indicated by pink bars.

References

Gruber,A.R. *et al.* (2008) The Vienna RNA websuite, *Nucleic Acids Res.*, **36**, W70-74.

Hofacker,I.L. (2003) Vienna RNA secondary structure server, *Nucleic Acids Res.*, **31**, 3429-3431.
