# Supplementary material for: RNA editing of nuclear transcripts in Arabidopsis thaliana
Source: BMC Genomics. 2010 Dec 2;11(Suppl 4):S12. doi: 10.1186/1471-2164-11-S4-S12 (PMC3005917; doi:10.1186/1471-2164-11-S4-S12)
Supplement: Additional file 4 — Data S4 Results of searching for conserved elements surrounding the editing sites in protein-coding genes. The results of three replicates of control analysis are included. [file 1471-2164-11-S4-S12-S4.zip › 13-additional-file4/Data S4.zip!/Data S4/A-U.pdf]

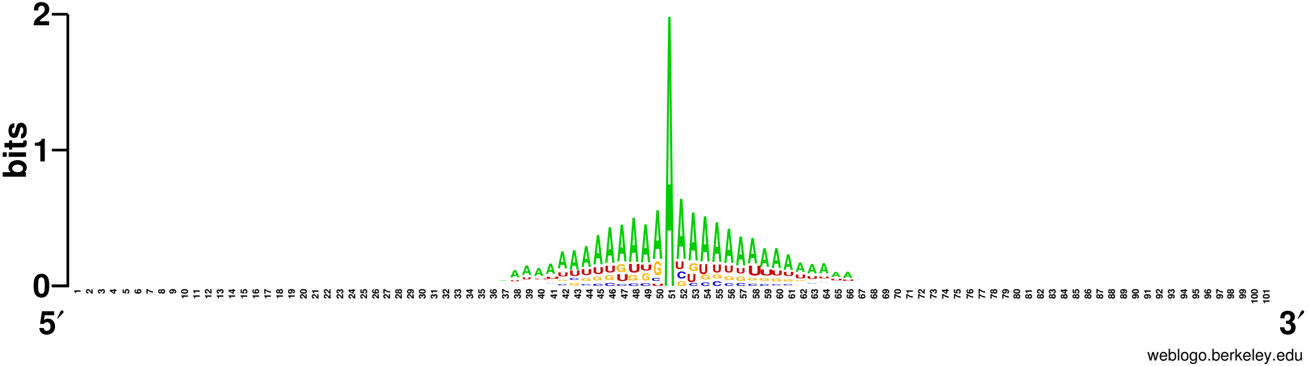
bits
2
1
0
1 2 3 4 5 6 7 8 9 10 11 12 13 14 15 16 17 18 19 20 21 22 23 24 25 26 27 28 29 30 31 32 33 34 35 36 37 38 39 40 41 42 43 44 45 46 47 48 49 50 51 52 53 54 55 56 57 58 59 60 61 62 63 64 65 66 67 68 69 70 71 72 73 74 75 76 77 78 79 80 81 82 83 84 85 86 87 88 89 90 91 92 93 94 95 96 97 98 99 100 101
5′
3′
weblogo.berkeley.edu
