# Supplementary material for: RNA editing of nuclear transcripts in Arabidopsis thaliana
Source: BMC Genomics. 2010 Dec 2;11(Suppl 4):S12. doi: 10.1186/1471-2164-11-S4-S12 (PMC3005917; doi:10.1186/1471-2164-11-S4-S12)
Supplement: Additional file 4 — Data S4 Results of searching for conserved elements surrounding the editing sites in protein-coding genes. The results of three replicates of control analysis are included. [file 1471-2164-11-S4-S12-S4.zip › 13-additional-file4/Data S4.zip!/Data S4/C-G.pdf]

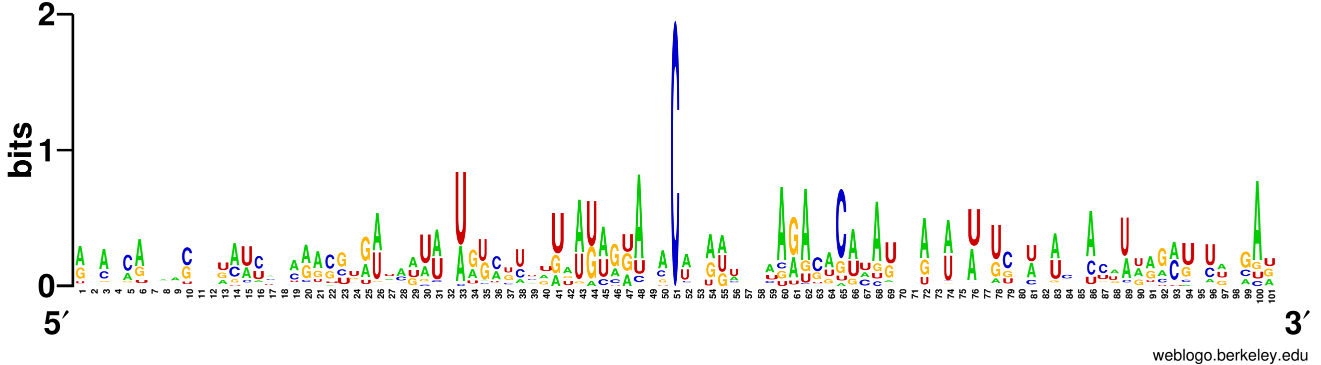

bits
5′
3′
weblogo.berkeley.edu
